# Real-World Drug Retention and Safety of Ustekinumab in Patients With Inflammatory Bowel Disease in Qatar: A Retrospective Cohort Study

**DOI:** 10.7759/cureus.111855

**Published:** 2026-06-30

**Authors:** Abdulwahab Hamid, Ahmed Badi, Ahmad Ayash, Yahaya Bensuleiman, Souraia Mezhoud, Mohammed Abderahim Hassan, Alia Battah, Munir Abu Ageila, Abdurahman Kday, Muneera Almohannadi

**Affiliations:** 1 Gastroenterology and Hepatology, Hamad Medical Corporation, Doha, QAT; 2 College of Medicine, Qatar University, Doha, QAT; 3 Internal Medicine, Hamad Medical Corporation, Doha, QAT; 4 Internal Medicine Residency Program, Medical Education, Hamad Medical Corporation, Doha, QAT

**Keywords:** crohn's disease, drug retention, inflammatory bowel disease, real-world evidence, ulcerative colitis, ustekinumab

## Abstract

Introduction: Inflammatory bowel disease (IBD), encompassing Crohn's disease (CD) and ulcerative colitis (UC), is an increasingly recognized condition in the Middle East and North Africa (MENA) region, yet real-world data on advanced therapies from this population remain scarce. Ustekinumab, a fully human monoclonal antibody targeting the shared p40 subunit of interleukin-12 and interleukin-23, has demonstrated efficacy and safety in pivotal randomized controlled trials for both CD and UC. However, its real-world performance in MENA populations has not been well characterized. This study aimed to evaluate the real-world drug retention, safety profile, and predictors of treatment discontinuation of ustekinumab in patients with IBD at two major referral centers in Qatar.

Methods: This was a retrospective, observational cohort study conducted at two IBD referral centers in Qatar (the Ambulatory Care Center and Al Khor Hospital, Hamad Medical Corporation). Adult patients (≥18 years) with a confirmed diagnosis of CD or UC who received at least one induction dose of ustekinumab were included. The primary outcome was drug retention at 6, 12, and 24 months. Secondary outcomes included the safety profile. The study was approved by the Medical Research Committee of Hamad Medical Corporation (MRC-01-25-1395).

Results: A total of 345 episodes were included (216 CD, 129 UC). Overall retention at 6, 12, and 24 months was 92.2%, 83.9%, and 77.8%, respectively. CD exceeded UC at all time points; the difference reached statistical significance at six months (95.4% vs. 86.8%; p = 0.011) but attenuated at 12 months (86.5% vs. 79.5%; p = 0.163) and 24 months (81.0% vs. 72.4%; p = 0.087). Discontinuation occurred in 79 episodes (22.9%); primary non-response (10.7%) and secondary loss of response (10.1%) were the main causes. Adverse events were documented in 14 episodes (4.1%); serious adverse events in 5 (1.4%). No malignancies or deaths were recorded. Shorter pre-treatment disease duration was a consistent predictor of discontinuation across all time points (6 months: p = 0.046; 12 months: p = 0.028; 24 months: p = 0.045). Disease type also met the significance threshold at six months (p = 0.011), with patients with UC demonstrating higher early discontinuation rates than those with CD.

Conclusions: In this retrospective cohort study, ustekinumab demonstrated high drug retention and an acceptable safety profile in a predominantly Arab IBD population in Qatar. Shorter disease duration prior to ustekinumab initiation was associated with treatment discontinuation across multiple time points, while patients with CD showed higher retention than those with UC at six months (p = 0.011). These findings suggest that disease type and timing of biologic initiation may be associated with ustekinumab persistence in this population. Further prospective studies are needed to confirm these observations in MENA populations with IBD.

## Introduction

Inflammatory bowel disease (IBD), which primarily encompasses Crohn's disease (CD) and ulcerative colitis (UC), is a chronic, relapsing-remitting inflammatory condition of the gastrointestinal tract. Historically considered a disease of the Western world, IBD has evolved into a global health challenge with a rapidly rising incidence and prevalence in newly industrialized regions, including the Middle East and North Africa (MENA) [1]. Recent epidemiological data indicate that the age-standardized prevalence of IBD in the MENA region has increased significantly over the past three decades, with countries in the Gulf region, such as Qatar, reporting some of the highest regional burdens [2,3]. This epidemiological shift, likely driven by rapid urbanization, dietary changes, and environmental factors, poses a substantial and growing challenge to regional healthcare systems [4].

Despite significant advancements in the therapeutic landscape of IBD, a substantial proportion of patients with moderate to severe disease either fail to respond initially (primary non-response) or lose response over time (secondary loss of response) to conventional therapies and first-line biologics, such as anti-tumor necrosis factor (anti-TNF) agents [5]. The management of these biologic-experienced patients remains a complex clinical challenge, necessitating the use of alternative therapeutic mechanisms to induce and maintain long-term remission [6].

Ustekinumab is a fully human IgG1κ monoclonal antibody that targets the p40 subunit shared by interleukin (IL)-12 and IL-23, thereby inhibiting key inflammatory pathways implicated in the pathogenesis of IBD [7]. The efficacy and safety of ustekinumab for inducing and maintaining remission in moderate to severe IBD were robustly established in the pivotal UNITI and UNIFI clinical trial programs [8,9]. Furthermore, long-term extension studies have demonstrated a favorable safety profile for ustekinumab, with low rates of serious infections and malignancies over up to five years of follow-up [10].

While randomized controlled trials provide foundational evidence for drug efficacy, real-world data are essential to validate these findings in broader, more heterogeneous patient populations encountered in routine clinical practice [11]. Several real-world observational studies from Western cohorts have reported high rates of clinical effectiveness and durable treatment persistence (drug survival) with ustekinumab, even among patients with prior exposure to multiple advanced therapies [12,13]. However, real-world evidence regarding the use, effectiveness, and safety of ustekinumab in Middle Eastern populations remains sparse [14]. Given the unique demographic characteristics, genetic backgrounds, and emerging disease phenotypes in this region, local data are crucial for optimizing IBD management strategies [15]. Therefore, this retrospective study aimed to evaluate the real-world drug retention and safety profile of ustekinumab in a diverse cohort of patients with IBD in Qatar.

## Materials and methods

Study design and setting

This was a retrospective, observational cohort study conducted across two major IBD care centers in Qatar: The Ambulatory Care Center and Al Khor Hospital. These facilities serve as the primary referral centers for the management of IBD in the country. The study was designed and reported in accordance with the Strengthening the Reporting of Observational Studies in Epidemiology (STROBE) guidelines [16].

Study population

The study included adult patients (aged 18 years and older) with a confirmed diagnosis of IBD based on standard clinical, endoscopic, radiological, and histological criteria. To be eligible for inclusion, patients must have received at least an induction dose of ustekinumab for the treatment of IBD between January 2019 and March 2025 at any of the participating centers. Patients were required to have a minimum potential follow-up period of six months following the initiation of ustekinumab, unless the treatment was discontinued earlier due to adverse events or primary non-response. Patients who received ustekinumab for indications other than IBD, those participating in concurrent clinical trials, and individuals with incomplete medical records precluding outcome assessment were excluded from the analysis.

Data collection and variables

Data were retrospectively extracted from the Cerner electronic medical records system, pharmacy dispensing logs, and laboratory information systems using a standardized electronic data collection form. Two trained researchers with experience in IBD care performed the data extraction to ensure consistency and accuracy. To protect patient confidentiality, all data were de-identified and assigned unique study codes before analysis.

The collected variables included baseline demographic characteristics (age at ustekinumab initiation, sex, nationality, ethnicity, and smoking status) and disease-specific parameters. Disease phenotype was categorized according to the Montreal classification system, detailing disease location and behavior for CD and disease extent and severity for UC [17]. Additionally, the presence of perianal disease in patients with CD and the duration of IBD before ustekinumab initiation were recorded.

Outcomes and definitions

The primary outcome of this study was drug retention (persistence) of ustekinumab, defined as the continuation of therapy at 6, 12, and 24 months after the initial induction dose. Drug retention serves as a reliable surrogate marker for overall treatment success in real-world settings, as it encompasses both clinical effectiveness and patient tolerability [18]. Reasons for treatment discontinuation were categorized as primary failure (lack of initial response), secondary failure (loss of response after initial improvement), adverse events, or other reasons (e.g., loss to follow-up).

Secondary outcomes included the evaluation of the safety profile of ustekinumab. Safety was assessed by reviewing clinical notes and hospital admission records for any documented adverse events during the treatment period. Special attention was given to serious adverse events, which were defined as events resulting in hospitalization, severe infections requiring intravenous antibiotics, malignancies, or death.

Statistical analysis

Statistical analyses were performed to evaluate drug retention and identify potential predictors of treatment discontinuation. Descriptive statistics were used to summarize baseline characteristics; continuous variables were presented as means with standard deviations or medians with interquartile ranges, depending on data distribution, while categorical variables were expressed as frequencies and percentages.

Time-to-event analysis for ustekinumab discontinuation was conducted using the Kaplan-Meier method. Differences in drug retention between subgroups (e.g., CD vs. UC) were compared using the log-rank test at each time point (6, 12, and 24 months). A Cox proportional hazards regression was prespecified to identify independent predictors of treatment discontinuation. However, this analysis could not be performed at the 12- and 24-month time points because an insufficient number of variables met the pre-specified entry threshold of p < 0.10 on univariate testing. At the six-month time point, two variables, disease type and disease duration prior to ustekinumab initiation, met this threshold, and a multivariable logistic regression model was therefore constructed at that time point. Univariate analyses at all three time points are reported. Statistical analyses were performed using IBM SPSS Statistics for Windows, Version 29.0 (Released 2022; IBM Corp., Armonk, NY, USA). A two-tailed p-value of <0.05 was considered statistically significant.

Ethical considerations

The study protocol was reviewed and approved by the Institutional Review Board (IRB) of the Medical Research Center at Hamad Medical Corporation (MRC-01-25-778). Given the retrospective, observational nature of the study and the use of de-identified data, a waiver of informed consent was granted by the IRB. The study was conducted in strict adherence to the ethical principles outlined in the Declaration of Helsinki and complied with local data protection regulations.

## Results

Study population and baseline characteristics

A total of 345 ustekinumab treatment episodes were identified across the two participating IBD centers. Of these, 216 episodes (62.6%) occurred in patients with CD and 129 (37.4%) in patients with UC. The mean age at ustekinumab initiation was 36.0 ± 12.5 years (median 35.0 years, IQR 26.0-44.0, range 18-71 years), with a similar age distribution between the CD (mean 35.8 ± 12.9 years) and UC (mean 36.5 ± 11.7 years). Males constituted the majority of the cohort (197 patients, 57.1%), and this proportion was comparable between CD (57.9%) and UC (55.8%) groups. Ethnically, the cohort was predominantly of Arab origin (264 episodes, 76.5%), reflecting the demographic composition of the study setting, followed by Asian (58, 16.8%) and White (23, 6.7%) backgrounds. The majority of patients were non-smokers (259 episodes, 75.1%), while 45 (13.0%) were active smokers and 28 (8.1%) were former smokers; smoking status was not documented in 13 episodes (3.8%). The median disease duration prior to ustekinumab initiation was 60.0 months (IQR 25.0-120.0), with data missing for two patients. Patients with UC had a numerically longer pre-treatment disease duration (median 72.0 months, IQR 36.0-132.0) compared to patients with CD (median 58.0 months, IQR 24.0-117.0).

Regarding CD phenotype, ileocolonic disease (L3) was the most prevalent location (106/216, 49.1%), followed by terminal ileal disease (L1, 78/216, 36.1%), with colonic (L2) and upper gastrointestinal involvement (L4) each accounting for 7.4% of cases. Stricturing behavior (B2) was the most common disease behavior (106/216, 49.1%), followed by penetrating (B3, 55/216, 25.5%) and inflammatory (B1, 54/216, 25.0%) phenotypes. Perianal disease was present in 63 CD episodes (29.2%). Among UC patients, extensive colitis (E3) was the predominant extent (75/129, 58.1%), followed by left-sided disease (E2, 46/129, 35.7%), pancolitis with backwash ileitis (E4, 5/129, 3.9%), and proctitis (E1, 3/129, 2.3%). Disease severity at the time of ustekinumab initiation was classified as moderate in 55 UC patients (42.6%) and severe in 54 (41.9%), with mild disease in 17 (13.2%) and undocumented severity in 3 (2.3%). Full baseline characteristics stratified by disease type are presented in Table 1.

**Table 1:** Baseline demographic and disease characteristics of the study cohort, stratified by disease type. Montreal classification used throughout. Disease duration data were missing for two episodes. IQR: interquartile range; CD: Crohn's disease; UC: ulcerative colitis; GI: gastrointestinal.

Variable	Overall (n = 345)	CD (n = 216)	UC (n = 129)
Demographic characteristics			
Age at initiation (years), mean ± SD	36.0 ± 12.5	35.8 ± 12.9	36.5 ± 11.7
Median (IQR)	35.0 (26.0–44.0)	34.0 (24.0–45.0)	36.0 (28.0–44.0)
Range	18–71	18–71	18–71
Male sex, n (%)	197 (57.1%)	125 (57.9%)	72 (55.8%)
Female sex, n (%)	148 (42.9%)	91 (42.1%)	57 (44.2%)
Ethnicity, n (%)			
Arab	264 (76.5%)	172 (79.6%)	92 (71.3%)
Asian	58 (16.8%)	33 (15.3%)	25 (19.4%)
White	23 (6.7%)	11 (5.1%)	12 (9.3%)
Smoking status, n (%)			
Never smoked	259 (75.1%)	158 (73.1%)	101 (78.3%)
Active smoker	45 (13.0%)	34 (15.7%)	11 (8.5%)
Ex-smoker	28 (8.1%)	20 (9.3%)	8 (6.2%)
Not reported	13 (3.8%)	4 (1.9%)	9 (7.0%)
Disease characteristics			
Disease duration before ustekinumab, months			
Median (IQR)	60.0 (25.0–120.0)	58.0 (24.0–117.0)	72.0 (36.0–132.0)
Missing data, n	2	-	-
CD: Montreal location, n (%)			
L1 (terminal ileum)	78 (36.1%)	78 (36.1%)	UC
L2 (colonic)	16 (7.4%)	16 (7.4%)	
L3 (ileocolonic)	106 (49.1%)	106 (49.1%)	
L4 (upper GI modifier)	16 (7.4%)	16 (7.4%)	
CD: Montreal behavior, n (%)			
B1 (non-stricturing, non-penetrating)	54 (25.0%)	54 (25.0%)	UC
B2 (stricturing)	106 (49.1%)	106 (49.1%)	
B3 (penetrating)	55 (25.5%)	55 (25.5%)	
Perianal disease, n (%)	63 (29.2%)	63 (29.2%)	
UC: Montreal extent, n (%)			
E1 (proctitis)	3 (2.3%)	CD	3 (2.3%)
E2 (left-sided)	46 (35.7%)		46 (35.7%)
E3 (extensive/pancolitis)	75 (58.1%)		75 (58.1%)
E4 (pancolitis with backwash ileitis)	5 (3.9%)		5 (3.9%)
UC: Disease severity, n (%)			
Mild	17 (13.2%)	CD	17 (13.2%)
Moderate	55 (42.6%)		55 (42.6%)
Severe	54 (41.9%)		54 (41.9%)
Not documented	3 (2.3%)		3 (2.3%)

Drug retention

Overall drug retention rates at 6, 12, and 24 months were 92.2% (318/345), 83.9% (287/342), and 77.8% (267/343), respectively. Three patients were censored at 12 months (one lost to follow-up) and two at 24 months, leaving 342 and 343 evaluable patients at those respective time points. When stratified by disease type, CD demonstrated higher retention rates than UC across all time points (Table 2). At 6 months, retention was 95.4% (206/216) for CD vs. 86.8% (112/129) for UC (p = 0.011); at 12 months, 86.5% (186/215) vs. 79.5% (101/127) (p = 0.163); and at 24 months, 81.0% (175/216) vs. 72.4% (92/127) (p = 0.137). The difference between disease types reached statistical significance at six months (p = 0.011), reflecting the higher rate of primary non-response in UC, but attenuated at 12 and 24 months as secondary loss of response became the predominant mode of failure in both groups. Cumulative drug retention rates at each time point are summarized in Table 2.

**Table 2:** Ustekinumab drug retention rates at 6, 12, and 24 months, by disease type. Denominators reflect evaluable episodes at each time point after accounting for censored observations. p = 0.087 for CD vs. UC comparison at 24 months (log-rank test, χ²(1) = 2.927). CD: Crohn's disease; UC: ulcerative colitis; n: number of patients retained at each time point.

Time point	Overall, n/N (%)	CD, n/N (%)	UC, n/N (%)	p-value (CD vs. UC)
6 months	318/345 (92.2)	206/216 (95.4)	112/129 (86.8)	0.011
12 months	287/342 (83.9)	186/215 (86.5)	101/127 (79.5)	0.163
24 months	267/343 (77.8)	175/216 (81.0)	92/127 (72.4)	0.137

At the six-month time point, both disease type (p = 0.011) and disease duration (p = 0.046) met the pre-specified entry threshold (p < 0.10) for multivariable analysis, suggesting that a multivariable logistic regression incorporating these two covariates is warranted and is presented as a supplementary analysis.

Reasons for treatment discontinuation

A total of 79 treatment patients (22.9%) were discontinued during the observation period. The most common reasons were primary non-response, occurring in 37 patients (10.7%), and secondary loss of response, in 35 patients (10.1%). Treatment was stopped due to adverse events in six episodes (1.7%), and one episode (0.3%) was lost to follow-up.

Stratified by disease type, UC had a numerically higher rate of total discontinuation (36/129, 27.9%) compared to CD (43/216, 19.9%) (p = 0.164). Primary non-response was more frequent in UC (21/129, 16.3%) than in CD (16/216, 7.4%) (p = 0.062), whereas secondary loss of response was comparably distributed (CD 9.3%, UC 11.6%; p = 0.705). All six discontinuations attributed to adverse events occurred in the CD cohort (2.8%), with no adverse event-related discontinuations observed in the UC group (p = 0.537). The full breakdown is presented in Table 3.

**Table 3:** Reasons for ustekinumab treatment discontinuation, stratified by disease type. Percentages were calculated against the total number of treatment episodes per disease group (CD, n = 216; UC, n = 129). Adverse events as a reason for discontinuation encompass only episodes in which the treating clinician explicitly attributed cessation to drug-related toxicity. Chi-squared test or Fisher's exact test was used to compare CD vs. UC for each discontinuation reason, as appropriate. Test statistics and p-values are provided in the test statistic column. CD: Crohn's disease; UC: ulcerative colitis.

Reason for discontinuation	Overall, n (%)	CD, n (%)	UC, n (%)	Test statistic (CD vs. UC)
Primary non-response	37 (10.7)	16 (7.4)	21 (16.3)	χ²(1) = 6.639, p = 0.010
Secondary loss of response	35 (10.1)	20 (9.3)	15 (11.6)	χ²(1) = 0.497, p = 0.481
Adverse events	6 (1.7)	6 (2.8)	0 (0.0)	Fisher's exact, p = 0.088
Lost to follow-up	1 (0.3)	1 (0.5)	0 (0.0)	Fisher's exact, p = 1.000
Total discontinued	79 (22.9)	43 (19.9)	36 (27.9)	χ²(1) = 2.927, p = 0.087

Safety profile

According to our cohort, Ustekinumab demonstrated a favorable safety profile. Adverse events were documented in 14 treatment episodes (4.1%). Of these, five (1.4%) were classified as SAEs leading to treatment discontinuation: an infusion-related allergic reaction (urticaria, chest tightness, and back pain) occurring at the time of the first intravenous induction dose, recurrent sepsis, herpes zoster reactivation, recurrent perianal abscess, and chronic neutropenia. All five SAEs occurred in CD patients.

The remaining nine adverse events (2.6% of all episodes) were managed conservatively without necessitating treatment cessation. These included two episodes of transient liver function test derangement (one CD, one UC), pyelonephritis, iron-deficiency anemia, a combination of iron-deficiency anemia and arthralgia, arthralgia alone, a transient psoriasiform skin rash, mild nausea, and a cluster of mild systemic symptoms comprising nausea, postprandial abdominal discomfort, and fatigue. All episodes with non-SAEs continued with ustekinumab maintenance therapy. No malignancies or deaths attributed to ustekinumab were recorded during the observation period. A detailed listing of all documented adverse events is provided in Table 4.

**Table 4:** Documented adverse events during ustekinumab treatment. "Discontinued" denotes that ustekinumab was permanently stopped as a direct consequence of the adverse event. Events listed as "Continued" indicate that the adverse event was managed without treatment cessation. The pyelonephritis episode was subsequently discontinued due to secondary loss of response, and the psoriasiform rash episode was discontinued due to primary non-response. SAE: serious adverse event; CD: Crohn's disease; UC: ulcerative colitis.

Adverse event	Disease	Outcome	Comment
Allergic reaction to ustekinumab infusion (skin rash, chest tightness, back pain)	CD	Discontinued	Treatment stopped at induction
Recurrent sepsis	CD	Discontinued	Treatment stopped; SAE
Herpes zoster	CD	Discontinued	Treatment stopped; SAE
Recurrent perianal abscess	CD	Discontinued	Treatment stopped; SAE
Chronic neutropenia	CD	Continued	Managed; treatment maintained
Pyelonephritis	CD	Continued	Managed; secondary failure (separate)
Liver function test derangement	CD	Continued	Monitored; treatment maintained
Liver function test derangement	CD	Continued	Monitored; treatment maintained
Transient psoriasiform rash	UC	Continued	Resolved; primary failure (separate)
Anemia (iron-deficiency)	CD	Continued	Supplementation; treatment maintained
Iron-deficiency anemia and joint pain	CD	Continued	Managed; treatment maintained
Joint pain	UC	Continued	Managed; treatment maintained
Mild nausea	CD	Continued	Symptom managed; treatment maintained
Nausea, postprandial pain, fatigue	CD	Continued	Managed; treatment maintained

Predictors of treatment discontinuation

Univariate analyses were performed to identify factors associated with ustekinumab discontinuation at 6, 12, and 24 months among evaluable episodes (n = 345, 341, and 343, respectively). Disease type (CD vs. UC), sex, age at initiation, smoking status, disease duration prior to treatment, and the presence of perianal disease (in CD) were examined as potential predictors at each time point.

Disease duration prior to ustekinumab initiation was the only variable to reach statistical significance as a predictor of treatment discontinuation across multiple time points. Episodes that were subsequently discontinued had a significantly shorter median pre-treatment disease duration compared to those that were retained at both six months (36.0 months, IQR 11.0-88.5 vs. 60.0 months, IQR 27.8-120.0; p = 0.046) and 12 months (36.0 months, IQR 12.2-117.0 vs. 60.0 months, IQR 28.0-120.0; p = 0.028) and at 24 months (36.0 months, IQR 16.5-96.0 vs. 60.0 months, IQR 25.0-120.0; p = 0.045, Mann-Whitney U test). This consistent finding across all three time points reinforces the observation that shorter pre-treatment disease duration is associated with a higher probability of treatment failure, potentially reflecting earlier-line biologic exposure or less immunologically selected disease.

Disease type showed a statistically significant association with discontinuation at six months, with UC demonstrating a higher discontinuation rate than CD (12.7% vs. 5.0%; p = 0.011). This difference attenuated and did not reach statistical significance at 12 months (19.5% vs. 13.8%; p = 0.163) or 24 months (27.6% vs. 19.0%; p = 0.087). The early divergence at six months, driven predominantly by primary non-response in UC, suggests that disease type is most influential in the initial treatment response phase. Sex (6 months: p = 0.064; 12 months: p = 0.350; 24 months: p = 0.823), age (6 months: p = 0.082; 12 months: p = 0.146; 24 months: p = 0.368), smoking status (6 months: p = 0.876; 12 months: p = 0.377; 24 months: p = 0.571), and the presence of perianal disease in CD patients (6 months: p = 0.181; 12 months: p = 0.521; 24 months: p = 0.348) were not significantly associated with treatment discontinuation at any time point. Given that disease type met the pre-specified threshold of p < 0.10 at six months, alongside disease duration (p = 0.046), a multivariable logistic regression model at six months is feasible and is presented below. Results of the univariate analyses across all three time points are presented in Table 5.

**Table 5:** Univariate analysis of predictors of ustekinumab discontinuation at 6, 12, and 24 months (n = 343 evaluable episodes). Categorical variables were compared by the chi-squared test; continuous variables were compared by the Mann-Whitney U test. Perianal disease analysis restricted to CD episodes only (n = 216). Disease duration data were missing for two episodes (excluded from that analysis). The "non-smoker/ex-smoker" group combines never-smoked and ex-smoker categories; 13 episodes with unreported smoking status were excluded from that comparison. p-values are reported separately for six-month, 12-month, and 24-month time points. At six months, n = 345 evaluable episodes; at 12 months, n = 341; at 24 months, n = 343. Age data missing for two episodes (excluded from age analysis at all time points). *Statistically significant at p < 0.05. IQR: interquartile range; CD: Crohn's disease; UC: ulcerative colitis.

Variable	Discontinued, n/N (%)	Retained, n/N (%)	p-value (6 months)	p-value (12 months)	p-value (24 months)
Disease type					
CD	41/216 (19.0%)	175/216 (81.0%)	0.011*	0.163	0.087
UC	35/127 (27.6%)	92/127 (72.4%)	0.011*	0.163	0.087
Sex					
Male	44/193 (22.8%)	149/193 (77.2%)	0.064	0.350	0.823
Female	32/151 (21.2%)	119/151 (78.8%)	0.064	0.350	0.823
Smoking status					
Active smoker	8/45 (17.8%)	37/45 (82.2%)	0.876	0.377	0.571
Non-smoker/ex-smoker	68/298 (22.8%)	230/298 (77.2%)	0.876	0.377	0.571
Disease duration before ustekinumab					
Discontinued, median (IQR)	36.0 months (16.5–96.0)	-	0.046*	0.028*	0.045*
Retained, median (IQR)	-	60.0 months (25.0–120.0)	0.046*	0.028*	0.045*
Age at Initiation					
Discontinued, median	32.5 years	-	0.082	0.146	0.368
Retained, median	-	36.0 years	0.082	0.146	0.368
Perianal disease (CD only)					
Perianal disease present	9/63 (14.3%)	54/63 (85.7%)	0.181	0.521	0.348
Perianal disease absent	32/153 (20.9%)	121/153 (79.1%)	0.181	0.521	0.348

## Discussion

Principal findings

This retrospective, two-center real-world study from Qatar represents, to our knowledge, one of the largest reported cohorts of ustekinumab use for IBD in the MENA region, encompassing 345 ustekinumab treatment episodes in patients with either CD or UC. The study demonstrates that ustekinumab achieves high and durable drug retention in a predominantly Arab, real-world clinical population, with overall retention rates of 92.2%, 83.9%, and 77.8% at 6, 12, and 24 months, respectively. Patients with CD exhibited consistently higher retention than those with UC across all time points. This difference reached statistical significance at six months (p = 0.011) but attenuated and did not reach significance at 12 months (p = 0.163) or 24 months (p = 0.087), suggesting that the CD-UC divergence is most pronounced during the early treatment phase. The safety profile was favorable, with adverse events documented in only 4.1% of treatment episodes and SAEs leading to discontinuation occurring in 1.4%. Shorter pre-treatment disease duration emerged as a consistent and statistically significant predictor of treatment discontinuation across all three time points (6 months: p = 0.046; 12 months: p = 0.028; 24 months: p = 0.045). Taken together, these findings align broadly with and extend the growing body of international real-world evidence supporting the long-term effectiveness and tolerability of ustekinumab in IBD.

Retention in context

The 24-month overall drug retention rate of 77.8% observed in our cohort is consistent with, and in many instances exceeds, rates reported from contemporaneous international real-world studies. In the Spanish SUSTAIN study, one of the largest real-world investigations of ustekinumab in CD, 77% of 463 heavily biologic-experienced patients remained on therapy after a median follow-up of 15 months, with a discontinuation incidence rate of 18% per patient-year [19]. A Swedish prospective multicenter cohort (the PROSE study, n = 114) reported notably lower retention of 70% and 61% at weeks 52 and 104, respectively [20]; however, this cohort was enriched for patients who had failed two or more anti-TNF agents (51%), which likely reflects a more treatment-refractory population than our study cohort. The higher retention observed in our study may reflect, at least in part, a greater proportion of patients initiating ustekinumab as an earlier-line advanced therapy, a practice increasingly supported by current guidelines, alongside the generally younger age of our cohort (mean 36.0 years).

A large population-based Japanese database study encompassing over 200 CD patients initiating ustekinumab as first-line advanced therapy reported 12- and 24-month persistence rates of 91.3% and 80.4%, respectively [21]. These figures closely parallel our CD subgroup retention (86.5% and 81.0%). For UC, a Spanish real-world cohort of 108 refractory patients treated across four tertiary centers reported ustekinumab persistence of 91% at 24 weeks, 83% at 48 weeks, and 81% at 96 weeks [22]. While our UC retention figures are somewhat lower (86.8% at 6 months, 72.4% at 24 months), the comparison should be contextualized by differences in follow-up intervals and eligibility criteria. The Phoenix retrospective Japanese cohort study of 150 UC patients likewise reported a one-year cumulative persistence rate of 84.0% [23], within the range of our UC findings at the same point (79.5%). Across these international benchmarks, our data confirm that the drug retention achieved in our cohort population is consistent with the global real-world experience, supporting the generalizability of ustekinumab's therapeutic durability beyond the predominantly Western or East Asian cohorts that have dominated the published literature to date.

Differential retention in CD versus UC

Across every follow-up time point in our study, patients with CD demonstrated higher ustekinumab retention than those with UC (95.4% vs. 86.8% at 6 months; 86.5% vs. 79.5% at 12 months; 81.0% vs. 72.4% at 24 months). Notably, this difference reached statistical significance at six months (p = 0.011), driven predominantly by the higher rate of primary non-response in UC (16.3%) compared to CD (7.4%), consistent with the early immunological divergence between the two disease phenotypes in response to IL-12/IL-23 blockade. The difference attenuated at 12 months (p = 0.163) and 24 months (p = 0.087), suggesting that the CD-UC gap is most clinically relevant in the induction and early maintenance phase. This pattern aligns with evidence from several independent cohorts. The Australian PANIC (Persistence Australian National Inflammatory Bowel Disease Cohort) registry study, which included 2,499 patients with 8,219 person-years of follow-up, demonstrated that ustekinumab had significantly superior persistence in CD compared to anti-TNF agents (HR 1.79, 95% CI 1.32-2.38, p < 0.01), with 12-month CD persistence rates of 80.0%, higher than those recorded for UC in the same dataset [24]. Furthermore, a Taiwanese single-center cohort of vedolizumab-experienced IBD patients reported that CD was an independent predictor of 52-week treatment persistence (OR 7.15, 95% CI 1.76-29.0, p = 0.006) when comparing ustekinumab with anti-TNF therapy [13].

The relatively lower retention rates in our UC group may be mechanistically explained by the pathophysiology of ustekinumab's mechanism of action. Ustekinumab targets the IL-12/IL-23 p40 subunit, a pathway perhaps more centrally implicated in the Th1/Th17-driven inflammation characteristic of CD, whereas UC harbors a more complex immunological milieu in which Th2-mediated pathways and additional targets, such as IL-13, may play a more prominent role [7]. This could translate into a relatively attenuated treatment response in UC, a hypothesis supported by the numerically higher primary non-response rate observed in our UC cohort (16.3%) compared to CD (7.4%). Similar observations have been reported in Japanese real-world UC cohorts, where patients with prior anti-TNF and vedolizumab exposure showed primary response rates of only 34.5% and 24.1%, respectively [23], further reinforcing that refractory UC represents a uniquely challenging therapeutic milieu, even with ustekinumab.

Reasons for treatment discontinuation

Treatment was discontinued in 22.9% of all patients during the observation period, with primary non-response (10.7%) and secondary loss of response (10.1%) constituting the dominant causes. These figures fall well within the range reported across the international real-world literature. A systematic review and meta-analysis evaluating ustekinumab in real-world UC cohorts reported overall discontinuation rates ranging from 11.5% to 43.7%, with lack of effectiveness accounting for the majority of these at 28.4% [25], higher than the combined non-response rate of 27.9% observed in our UC group. The SUSTAIN study reported an annualized discontinuation incidence of 18% per patient-year in CD [19], broadly consistent with our CD discontinuation rate of 19.9% over 24 months. The higher primary non-response rate in UC (16.3%) vs. CD (7.4%) in our cohort is biologically plausible and clinically consistent with the differential response rates observed in the pivotal UNIFI trial, wherein a lower proportion of UC patients achieved clinical remission at week 8 compared to analogous time points in the UNITI CD study [8,9].

Notably, all six episodes discontinued due to adverse events occurred in the CD cohort, with no adverse event-related discontinuations in UC. While the reasons for this disease-specific distribution may relate to the higher proportion of immunosuppression-related complications in complex, biologic-experienced patients with CD or the greater prevalence of perianal and penetrating disease (B3 25.5%) in this group, which carries an inherently higher risk of infectious complications, this finding should be interpreted cautiously given the small absolute number of adverse-event-related discontinuations.

Safety profile

The favorable safety profile observed in our study, with adverse events documented in only 4.1% of episodes and SAEs leading to discontinuation in just 1.4%, is in accord with both pivotal trial data and accumulating real-world evidence. The IM-UNITI long-term extension study, which followed CD patients on ustekinumab for up to five years, reported comparable SAE rates per 100 patient-years in the ustekinumab arm vs. placebo (19.3 vs. 17.5), with serious infection rates of 3.9 vs. 3.4 per 100 patient-years [26]. The most recent pooled safety analysis encompassing five years of CD and four years of UC follow-up from the phase 2/3 ustekinumab program similarly demonstrated that overall adverse event rates and SAE rates were consistently lower in ustekinumab-treated patients than in those receiving placebo (347.47 vs. 482.41 and 18.85 vs. 29.39 per 100 patient-years, respectively) [27].

The five SAEs identified in our cohort (herpes zoster reactivation, recurrent sepsis, recurrent perianal abscess, chronic neutropenia, and an infusion-related allergic reaction) are consistent with the established safety signal class of ustekinumab and do not represent unexpected findings. Herpes zoster reactivation is a recognized adverse event with IL-12/IL-23 inhibitor therapy; the pooled safety analysis noted a modestly higher rate of herpes zoster in CD patients on ustekinumab compared to placebo [27]. The infusion-related allergic reaction observed at the time of the first intravenous induction dose in one patient aligns with the known class effect of intravenous biologic administration and has been characterized in post-marketing pharmacovigilance analyses of ustekinumab [28]. Crucially, no malignancies and no deaths attributable to ustekinumab were recorded during our observation period, which further corroborates the long-term safety reassurance provided by the IM-UNITI and UNIFI trial extension programs [26,27].

The nine adverse events managed without treatment cessation, encompassing liver function test derangement, arthralgia, anemia, and mild gastrointestinal symptoms, are consistent with the non-SAE spectrum described in real-world UC and CD cohorts. A Japanese real-world UC study (n = 150) reported an adverse event incidence of 6.4%, slightly higher than the 4.1% observed in our series, potentially reflecting differences in monitoring frequency or adverse event ascertainment [23]. The overall safety experience in our Qatari cohort lends further confidence to the tolerability of ustekinumab in a demographically distinct, predominantly Arab population, in whom pharmacogenomic differences in drug metabolism or immunological responsiveness have been postulated but remain insufficiently characterized.

Predictors of treatment discontinuation

Shorter pre-treatment disease duration was the only variable to reach statistical significance as a predictor of treatment discontinuation consistently across all three time points in our univariate analyses: at six months (median 36.0 months in discontinued episodes vs. 60.0 months in those retained; IQR 11.0-88.5 vs. 27.8-120.0; p = 0.046), at 12 months (median 36.0 vs. 60.0 months; IQR 12.2-117.0 vs. 28.0-120.0; p = 0.028), and at 24 months (median 36.0 vs. 60.0 months; IQR 16.5-96.0 vs. 25.0-120.0; p = 0.045, Mann-Whitney U test). The consistency of this finding across all time points substantially strengthens the conclusion that shorter pre-treatment disease duration is a robust predictor of ustekinumab treatment failure. This finding suggests that patients with a relatively shorter established disease trajectory, perhaps reflecting earlier-line biologic exposure, less immunological tolerance induction, or incompletely optimized prior therapy, may be at higher risk of treatment failure at any stage of follow-up. This observation is mechanistically plausible: patients with longer disease duration and multiple prior treatment failures may have a more selected, disease-resistant immune phenotype that paradoxically responds better to a novel mechanism of action such as IL-12/IL-23 blockade, or they may have already undergone extensive optimization of concomitant therapies.

The predictors of ustekinumab retention vary across real-world studies depending on the populations studied. The SUSTAIN study (CD, n = 463) identified prior intestinal surgery and concomitant corticosteroid use as independent predictors of discontinuation, while maintenance dosing interval (every 12 weeks rather than every 8 weeks) was paradoxically associated with lower discontinuation risk [19], variables not captured in this study. A Japanese real-world UC study identified high C-reactive protein levels at baseline as a significant risk factor for ustekinumab discontinuation [25], and the Phoenix cohort found that the chronic continuous UC disease course and prior anti-TNF plus vedolizumab co-exposure were negative predictive factors for early response [23]. A particularly important variable not systematically captured in this study is prior biologic exposure. Across the international real-world literature, biologic-naive patients and those receiving ustekinumab as a first advanced therapy consistently demonstrate higher treatment persistence than heavily pre-treated patients with multiple prior biologic failures [19,23]. Given the tertiary referral nature of both participating centers, a significant proportion of our cohort is likely to have had prior exposure to anti-TNF agents and/or vedolizumab, which would be expected to exert downward pressure on retention rates. The fact that our observed retention rates remain comparable to or exceed those of international cohorts despite this likely enrichment for biologic-experienced patients is therefore noteworthy and may reflect the relatively younger age of our cohort, the earlier adoption of ustekinumab as a second-line rather than third-line agent in recent years, or population-specific immunological factors. Future studies from this region should prospectively capture biologic exposure history to enable stratified analyses by treatment line. In this study, at the six-month time point, both disease duration (p = 0.046) and disease type (p = 0.011) met the pre-specified entry threshold of p < 0.10, fulfilling the criteria for entry into a multivariable logistic regression model. This represents an advance over the 12- and 24-month analyses, where only disease duration met this threshold. A multivariable model at six months incorporating these two variables would allow estimation of the independent contribution of each predictor while controlling for the other and is a natural next step for future analysis. At 12 and 24 months, the number of variables meeting the entry threshold remained insufficient for a robust multivariable model, underscoring the need for larger prospective regional cohorts with richer covariate data to fully characterize the predictors of long-term ustekinumab retention in this population.

Demographic and disease phenotype observations in a MENA population

Our cohort was predominantly of Arab ethnicity (76.5%), reflecting the demographic composition of Qatar's national patient population, and included a significant proportion of Asian patients (16.8%), consistent with the country's large South Asian expatriate community. The mean age at ustekinumab initiation of 36.0 years is younger than most Western ustekinumab cohorts; for example, the SUSTAIN study reported a mean baseline age of approximately 40 years [19], which may reflect the generally younger age of IBD onset described in MENA populations and may have implications for both disease progression and long-term treatment sequencing requirements [2,4].

Active smoking was more prevalent in the CD cohort (15.7%) than in the UC group (8.5%), a distribution consistent with the well-established divergent association of tobacco with IBD subtypes: smoking is a recognized risk factor for CD while exerting a putatively protective effect in UC. This observation is corroborated by epidemiological data from Lebanese and broader MENA IBD cohorts [15], as well as by a 2024 United States cohort study that confirmed active smoking was not associated with accelerated progression through biologic therapies in CD, despite being a well-established risk factor for relapse and surgical outcomes [29]. The finding that smoking status was not a significant predictor of ustekinumab discontinuation in our cohort (p = 0.571) aligns with this emerging literature.

With respect to disease phenotype, ileocolonic involvement (L3, 49.1%) and stricturing behavior (B2, 49.1%) were the predominant CD phenotypes, while extensive colitis (E3, 58.1%) dominated the UC group. The high prevalence of moderate-to-severe UC (41.9% severe at initiation) likely reflects the tertiary-referral nature of both participating centers and the policy of reserving ustekinumab for patients who have failed or are intolerant of at least one prior advanced therapy, although the absence of detailed prior biologic exposure data is acknowledged as a limitation of this study.

Limitations

This study carries some limitations inherent to its retrospective, observational design. First, drug retention was used as the primary outcome measure in lieu of objective measures of clinical, biochemical, or endoscopic remission. While retention is an established and widely accepted surrogate for overall treatment success in real-world IBD cohorts [18], it does not capture the proportion of patients who achieved deep remission or mucosal healing. Second, prior biologic exposure history was not systematically captured in the present analysis, precluding subgroup analyses stratified by biologic naivety or treatment line. This is an important limitation, as prior biologic exposure is one of the most consistently reported predictors of ustekinumab retention across the international literature [19,23], and its absence limits the interpretability of the predictor analysis and the comparability of retention rates with cohorts of known biologic exposure status. Third, while the six-month univariate analysis identified two variables meeting the pre-specified entry threshold (p < 0.10), disease type and disease duration, enabling a multivariable logistic regression at that time point, the 12- and 24-month analyses remained limited to univariate testing due to an insufficient number of qualifying variables, limiting the ability to identify independent predictors of longer-term discontinuation while controlling for confounders. Finally, although this is one of the first and largest real-world ustekinumab cohorts published from the MENA region, the study remains single country and may not capture the full ethnic and socioeconomic heterogeneity of the broader regional IBD population.

## Conclusions

In this retrospective cohort study, ustekinumab was associated with high drug retention and an acceptable safety profile in a predominantly Arab IBD population, with no treatment-attributable malignancies or deaths observed during follow-up. Patients with CD exhibited higher early drug retention than those with UC at six months (p = 0.011). Shorter disease duration prior to ustekinumab initiation was consistently associated with treatment discontinuation across multiple time points, suggesting a potential relationship between treatment timing and long-term persistence. These findings provide real-world data on ustekinumab use in the Middle East and contribute to the limited evidence available from populations that have historically been underrepresented in the global IBD literature.
